# User perspectives on critical factors for collaborative playlists

**DOI:** 10.1371/journal.pone.0260750

**Published:** 2022-01-05

**Authors:** So Yeon Park, Blair Kaneshiro

**Affiliations:** 1 Center for Design Research, Stanford University, Stanford, CA, United States of America; 2 Center for Computer Research in Music and Acoustics, Stanford University, Stanford, CA, United States of America; Northwestern University, UNITED STATES

## Abstract

Today, collaborative playlists (CPs) translate long-standing social practices around music consumption to enable people to curate and listen to music together over streaming platforms. Yet despite the critical role of CPs in digitally connecting people through music, we still understand very little about the needs and desires of real-world users, and how CPs might be designed to best serve them. To bridge this gap in knowledge, we conducted a survey with CP users, collecting open-ended text responses on what aspects of CPs they consider most important and useful, and what they viewed as missing or desired. Using thematic analysis, we derived from these responses the Codebook of Critical CP Factors, which comprises eight categories. We gained insights into which aspects of CPs are particularly useful—for instance, the ability for multiple collaborators to edit a single playlist—and which are absent and desired—such as the ability for collaborators to communicate about a CP or the music contained therein. From these findings we propose design implications to inform further design of CP functionalities and platforms, and highlight potential benefits and challenges related to their adoption in current music services.

## Introduction

Selecting and listening to music together are long-standing social activities. One such activity is music co-curation, which has a rich history spanning multiple use cases over the past century, from technologies and artifacts predating digital music collections (e.g., jukeboxes, mixtapes) to today’s usage of music streaming platforms. Among various methods through which users can curate music together on streaming platforms is co-editing of a collaborative playlist (CP), which is “a list of songs that multiple users have created using a digital platform” [1].

CP functionalities have been available on major commercial streaming platforms for over a decade, enabling users to socially curate and consume music in a manner similar to personal music curation. However, relatively little attention has been paid to CPs. In terms of platform implementation, only a few major streaming services such as Spotify (https://www.spotify.com/), Deezer (https://www.deezer.com/), and YouTube (https://www.youtube.com/) currently offer CP functionalities; to the best of our knowledge, these implementations are essentially personal playlists with co-editing functionalities that allow multiple users to add, delete, or reorder tracks [2]. Moreover, while the study of present-day music consumption is an established sub-field of music information retrieval (MIR) [3], most user research relating to music streaming platforms—e.g., characterizing tastes [4], recommending songs [5], or facilitating search [6]—focuses on individual usage. While the exact number of CP users are unknown, prior work has found that 58–80% of survey samples—consisting of Spotify users primarily in the US—were CP users [1, 9]. Despite these sizable proportions of CP users, studies of CPs and their usage are few in number compared to personal playlists [1, 7–9]. Consequently, our understanding of how CPs are used, and how streaming platforms can best support users in the social curation setting, is relatively lacking.

In 2020, shutdowns related to the COVID-19 pandemic forced many human-to-human interactions to become virtual [10]. As a result, a number of music-related activities have been impacted, including performing together [11], taking lessons [12], and attending concerts [13]. CPs, too, have received added attention at this time [14–16]. While the proliferation of virtual or socially distanced musical activities is currently a necessity, these trends may ultimately translate to long-term, fundamental changes in how we interact [17]. Thus there is a need, now more than ever, for well-designed social platforms—including those created for musical activities.

In this study we extend the literature on CPs by focusing specifically on user perceptions of CPs and their usage. As our main research question, we ask,

*What aspects of CPs and their experience are most important to users?*

We address this question by unpacking free-text responses from *N* = 70 real-world CP users, all of whom were found to have engaged with CPs using Spotify. Participants reported on (1) what they feel is most useful and (2) what they find missing from existing CP platforms. From thematic analysis of these responses, we derived the Codebook of Critical CP Factors, which comprises eight aspects of CPs and their usage. Based on the extent to which the responses collectively implicated—positively and negatively—each of the eight codebook categories, and on insights from related literature, we derived design implications to inform future design of CP platforms. Taken together, this study extends the body of user research in music information retrieval—and human-computer interaction more broadly—by providing much-needed insights into digitally mediated social music curation, while also informing real-world platform design.

## Related works

We provide background on music curation from prior literature on general music collections as well as CPs. As CPs are one way in which users engage with one another through music, we also expand upon social music activities. Finally, we relate CPs to social music prototypes and collaborative platforms, as findings from these studies may inform design of CP platforms.

### Music curation and playlists

Music curations have existed in many forms, from LP collections to mixtapes. Today, collections of songs are often organized digitally as playlists [18]. Playlists—generally assumed to be for personal use—can be created by users or provided by the streaming platform. They can be centered around specific themes or contexts, such as holidays [19] or a particular year [18], or accompany everyday activities such as working or exercising [18, 20, 21]. Playlists can serve as a static record of music originally added, or be updated continuously [18]. The ease of accessing playlists on streaming platforms enables users to curate music, share it publicly, and even gift music as digital mixtapes (https://developer.spotify.com/documentation/general/guides/working-with-playlists/). Services such as 8tracks (https://8tracks.com/duewets), Playlists.net (https://playlists.net/), and Art of the Mix (http://www.artofthemix.org/) enable users to share (i.e., let others listen to but not co-curate) playlists online.

Social music curation and consumption are carried out on streaming platforms as well. Playlists can now be curated by and shared among users [18, 21], with CP functionalities that enable multiple users to listen to or edit a playlist from multiple devices [2]. Following a preliminary study exploring evolution, usage, and perceptions around CPs [8], Park et al. introduced the CP Framework, which includes three purposes—Practical, Cognitive, and Social—that motivate real-world users to engage with these shared playlists [1]. Here, Practical purposes pertain to characteristics of the CP, curation process, and consumption contexts; Cognitive purposes relate to learning and discovery about music in the CP or about ones’ collaborators; and Social purposes pertain to sharing the playlist, sharing music more generally, or bonding and connecting with collaborators. More recently, Park & Kaneshiro conducted a mixed-methods investigation in order to characterize successful CPs and their usage, investigating factors such as who initiated the CP, with whom users engaged in CPs, and how they interacted with the CPs [8]. Experimental research further underscores social effects on music curation: Bauer & Ferwerda found that positive and negative judgments of simulated collaborators (i.e., bots) differentially affected participants’ curation decisions [22], while Park & Lee found that perceived ownership of CPs and their songs influenced engagement [9].

Even so, relatively little is known about CPs and their usage. The bulk of innovation on today’s music streaming platforms is geared toward personalization over socialization [5], and today’s CPs embody essentially what we term a “personal-plus” implementation—that is, a personal playlist interface with co-editing capabilities. Yet the ideal CP design may be something quite different. User studies on CPs have provided tangible first insights into users’ perspectives [1, 7, 8]; however, the potential of experiential reports to inform ideal design specifications of CPs is limited by current affordances of the CP platform.

### Social music activities

Selecting and consuming music is a pleasurable activity, whether undertaken alone or with others [23–25]. Curating music for others has evolved with technology over the past century, from dedicating songs to others over the radio [26] to the use of jukeboxes in social settings [27] and the rise of mixtapes and CDs [28, 29]. Other studies have investigated practices of music selection for shared in-person consumption—e.g., for road trips [30] and parties [31], or in the home [32].

As music collections migrated to digital formats such as iTunes and Napster in the early 2000s, studies of content sharing and curation over those platforms followed [24, 28]. In terms of social practices on commercial streaming platforms, Spinelli and colleagues conducted focus groups to derive a codebook comprising nine social practices and 24 influences; for example, group size, group dynamic, event/activity, and effort/engagement were found to affect music selection practices [33]. Social media, too, enables users to effortlessly share musical interests and links to the music itself [34], share sentiments about music [35], and connect with artists [36] and other fans [37–39]. Since 2020, COVID-19 restrictions have forcibly reshaped the ways in which people consume music together. For example, livestreamed virtual concerts fulfill some (but not all) of the social needs of concertgoers [40], while Tim’s Twitter Listening Party (https://timstwitterlisteningparty.com/) invites artists to live-tweet as they and their fans listen to one of their albums remotely yet synchronously; these events are thought to provide a new means for collective socialization and reminiscence around music [41].

### Social music prototypes

Field studies involving digital social music prototypes date back over 20 years. While prototype studies generally do not reflect real-world usage of widely used services, their insights can highlight specific affordances and features that cannot currently be observed on commercial platforms, and can thus potentially inform design implications for real-world CP platforms.

An early example, MusicFX, selected music to broadcast in a gym using a group preference agent [42]. Subsequent proximity-based systems designed to broadcast group playlists include Flytrap [43] and Adaptive Radio [44] systems; in these systems, music was selected for consumption in shared settings based upon listeners’ positive and negative preferences, respectively, while Jukola enacted a voting system for music selection in a bar [45] and Sound Pryer shared music among drivers in cars based on proximity [46]. Other systems were aimed toward consumption over mobile devices. For instance, the tunA system enabled users to browse playlists, bookmark songs, and message with nearby users [47], while Push!Music implemented a more tangible form of music sharing by permitting automatic copying or manual sharing of songs across users’ devices [48]. Social Playlist, aiming to support already-established relationships among users, provided “a shared playlist where members associate music from their personal library to their activities and locations” [49]. More recently, Kirk et al. introduced Pocketsong, which allowed users to observe what others were listening to, as well as “gift” snippets of songs to others [50]. Finally, MoodPic from Lehtiniemi et al. enabled users to collaboratively curate music by associating songs to “mood pictures” [51, 52].

Numerous valued (or requested, if not implemented) attributes of social music systems have emerged across these prototype studies. For instance, listening, sharing, and voting behaviors of others helped users discover music [45, 47, 48], learn about their friends [49], and even discover others with similar tastes [47, 52]. Such discoveries can lead to surprise and even serendipity, e.g., when encountering shared musical tastes [44] or songs contributed by others [49, 51]. In addition, social functionalities such as commenting, messaging, rating, and voting were usually viewed positively [45, 47, 49–52] and noted as ways to potentially strengthen existing social relationships [49] or form new ones [47], clarify song selections [49], and facilitate music discovery [52]. Finally, some users felt gratified knowing that others had listened to a song they contributed [49, 51].

Prototype studies also highlight challenges and complications that are unique to social scenarios. Lehtiniemi & Ojala reported varying, sometimes conflicting requests for collaborators’ editing control of a playlist [51]; others noted that a collaborative system could produce “simply an overflow of songs” [48] or bad songs in particular [49]. Despite positive aspects of social features noted above, some users worried that too much extramusical content in the platform could “reduce the main role of the music in the service” [51]. Finally, some users expressed an interest in knowing what others were listening to [48, 50, 51], while others felt shy about adding songs (e.g., because they did not know the collaborator [51] or collaborators’ tastes [50]) and noted that they might change their listening behaviors, should those behaviors be made visible to others [50].

### Other collaborative platforms

While CPs differ from other types of collaborative artifacts in critical ways [8], they may be broadly considered a form of collective content as defined by Olsson, in that they are “digital media content that is regarded as *commonly owned* as well as *jointly created* and *used*”, and “both a consequence of collaborative activities with content and […] a reason and motivator for collaborative activities to occur around content” [53]. Thus, other collaborative platforms may also provide insights relevant to user needs for CPs.

Numerous studies have investigated collaborations over digital platforms. Collaborative writing is prolifically studied in computer-supported cooperative work, and has led researchers to design for version control management [54, 55], annotations [55, 56], and access methods [57, 58]. Services such as Google Docs provide functionalities that enable synchronous co-editing (https://www.google.com/docs/about/), multiple access levels (i.e., view, suggest, edit) [59], and an easily accessible version history through which users can view others’ edits and revert to previous versions. Such functionalities can be beneficial to making contributions more visible, and thereby increase change awareness [60]; however, they can also bring about social conflict [61]. One study on collaborative authors has found that edits are made in socially conscious ways to mitigate such conflicts [62]. Other features, such as the display of who is currently viewing or editing the document, promote “workspace awareness”, an aspect of in-person collaboration which must be intentionally designed for in virtual settings [63, 64]. Finally, studies of co-editing on Wikipedia have identified collaborative elements underlying article quality [65], and have proposed user personas—such as “zealots” and “Good Samaritans”—as a means of elucidating the nature and value of different collaboration styles [66].

Online collaborations are also known to support social functions. Some online communities, such as special-interest groups, are formed with a social component in mind [53, 67]; when a technology platform and the collaborative contributions enacted therein combine effectively, the “snowball effect”—described by Olsson and colleagues as “reciprocal activity that maintains or increases content-related and social interaction” [67]—can be achieved.

A number of known challenges are identified in past research. Collaborations can be hindered by access—for example, if not all collaborators use the same platform [57], or if a cloud-based system does not permit offline editing, as was previously the case with Google Docs [68]. While collective content has the potential to enter a positive cycle of engagement [67], “social loafing”—the reduction in individuals’ efforts when working as part of a group [69–71]—is known to be a general issue in online communities [72–74]. As suggested in prior CP work [7, 9], uneven contributions and differing perceptions of ownership can lead to territoriality issues, which have also been noted around collaborative authoring in Wikipedia [75, 76] and co-curation in Pinterest [77].

## Methods

### Ethics statement

This study was approved by Stanford University’s Institutional Review Board. All participants confirmed their eligibility, and indicated informed consent by agreeing to conditions detailed in an Information Sheet (Waiver of Consent), prior to participating in the study.

### Survey design

We conducted a survey to better understand use cases around CPs from real-world users. In order to ground participants, we first provided the following definition of CPs from Park et al. [1]: “A list of songs that multiple users have created using a digital platform”. Then, the users answered various questions (not analyzed here) about their favorite CPs to further ground their CP experience in a specific use case. Finally, we asked users about “features” to enable them to think in a more tangible way, and to enable us to derive aspects that are necessary for positive collaborative experiences; these are the questions considered in the present analysis. These specific survey questions, to which CP users provided free-text responses, are as follows:

**Q1**: What features of a collaborative playlist are most important or useful to you?
**Q2**: What are some shortcomings that you see in today’s collaborative playlist platforms? What are some features that you believe would enhance the collaboration between you and your playlist collaborators?

While open-ended questions, as used here, are known to introduce challenges around the quality of responses and added analysis effort [78–83], they also allow respondents to elaborate on their thoughts [81] and express ideas that researchers may not have thought of as a priori response options [78, 79, 83]. Moreover, open-ended responses are deemed useful at initial stages of research to “[classify] the structure of a problem in all its details” [84], and are recommended “if it is not yet possible to clearly delimit the subject of inquiry, or if one expects new topics to emerge” [81]. As the present study is to the best of our knowledge the first exploration of user needs around CPs, we thus deemed the open-ended format to be appropriate.

### Analysis

Seeking to identify aspects of CPs that emerge from the data—including those that could be overlooked in the application of a pre-existing theoretical framework—we took an inductive approach to data analysis [85, 86]. Recent user research in MIR using such similar approaches has successfully characterized users of streaming services [87], user comments on SoundCloud [35], social practices surrounding streaming services [33], and purposes for engaging with CPs [1].

We analyzed responses across the two questions, as they collectively implicated shared aspects of CPs and their usage. After aggregating the responses, we used thematic analysis to manually group individual ideas expressed therein [86]. The themes that emerged from this analysis were grouped further under higher-level themes when possible (e.g., “Control Settings”, “Catalog Access”, and “Platform Access” were all grouped under “Access”). The final set of high-level groupings formed the eight categories of the Codebook of Critical CP Factors (Table 1). We subsequently documented the codebook with descriptions to aid in the coding process [88]. Once the codebook was finalized, we performed consensus coding over the full set of responses. Each free-text response could receive multiple codings; responses were coded at the category level as well as the sub-category level, when sub-categories existed. The two authors each independently coded all of the text responses, which yielded high inter-rater reliabilities (Krippendorff’s alpha: 0.989 for Q1, 0.985 for Q2). All discrepancies between coders were resolved through discussion.

**Table 1:** Codebook of Critical CP Factors, derived from thematic analysis of free-text responses delivered by CP users regarding which aspects of CPs are most useful and important, and which are desired or lacking.

**1. Access**. Pertaining to platform affordances for accessing and editing CPs: Settings to **control CP access**; music **catalog size and navigability**; or access to the **platform** itself. 1.1. Control settings 1.2. Catalog access 1.3. Platform access
**2. Content**. Pertaining to **characteristics or quality** of songs in the CP—e.g., their variety, diversity, or newness; the CP’s musical theme; or songs being reflective of all tastes or needing quality control—or to **display** content to users. 2.1. Content attributes & quality 2.2. Content display
**3. Initiation & Editing**. Pertaining to **making or creating** a CP, **editing** the CP (alone or with **multiple editors**), or accessing a **historical record** of edits. 3.1. Initiation 3.2. Editing artifact 3.3. Multiple editors 3.4. Record
**4. Consumption**. Pertaining to how users **listen to the CP**—whether alone or with others, synchronously or asynchronously, and to song order when listening (i.e., shuffling)—or to **analytics** on listening. 4.1. Music listening 4.2. Consumption analytics
**5. Communication**. Pertaining to the means by which users can **communicate** and **express sentiment** to one another about CPs and their content—e.g., via chat, comments, likes, and ratings.
**6. Discovery**. Pertaining to inwardly directed acts of **learning** or **receiving information** in the course of engaging with a CP—e.g., discovery of new **music**; learning about **collaborators’ musical tastes**; or discovering **new collaborators** or **new CPs** with which to engage. 6.1. Music discovery 6.2. Discovery of others’ music 6.3. Discovery of CPs & collaborators
**7. Social**. Pertaining to outwardly directed acts of **sharing** or **recommending** music to others, or to social **social connections** facilitated or signified by CP usage. 7.1. Share & recommend 7.2. Connect
**8. Engagement**. Pertaining to the extent and nature of user engagement with a CP—e.g., **longevity** of a CP collaboration, **awareness** of CPs in general or of a particular CP, **intrinsic process** of using a CP, or (inter-)personal **feelings** that help or hinder collaboration around a CP.

With few exceptions, responses to Q1 implicated positive perceptions, which we report as “Useful/Important” aspects of CPs and their usage; responses to Q2, reported as shortcomings or missing features, are framed as “Lacking/Desired” aspects. We report percentages of responses implicating each of the eight codebook categories (and their sub-categories, when applicable) as well as illustrative quotes identified by anonymized participant number (e.g., P12). The complete set of participant responses is provided as supplementary data in S1 Table.

### Participants

We collected responses from *N* = 70 CP users. This sample size of real-world users is on par with or greater than those reported in recent published CP research [1, 8]. Participants were recruited through various music-related groups on social media, listservs, and flyers. Participants ranged in age from 18 to 59 years (*M* = 24.2, *SD* = 7.8), 51% were female, and all used Spotify to engage in CPs. All participants were from the United States or a territory thereof. Responses were given anonymously; upon completing the study, participants were redirected to a separate form to input their email address to be entered into a raffle with 10% of winning a $10 Amazon gift card. All responses were collected in 2019, before the onset of COVID-19 shutdowns.

All but one participant reported at least one useful or important aspect of CPs (Q1), and all but two reported lacking or desired aspects (Q2). For Q1, text responses ranged in length from 1 to 58 words (*M* = 13.9, *SD* = 13.3 words). For Q2, where participants wrote about both shortcomings and desired features, responses ranged from 2 to 218 words in length (*M* = 26.7, *SD* = 29.5 words).

## Results

Our thematic analysis yielded the Codebook of Critical CP Factors, which consists of eight high-level categories. The eight high-level factors of the codebook, and their sub-categories where applicable, are summarized in Table 1.

**Fig 1:**
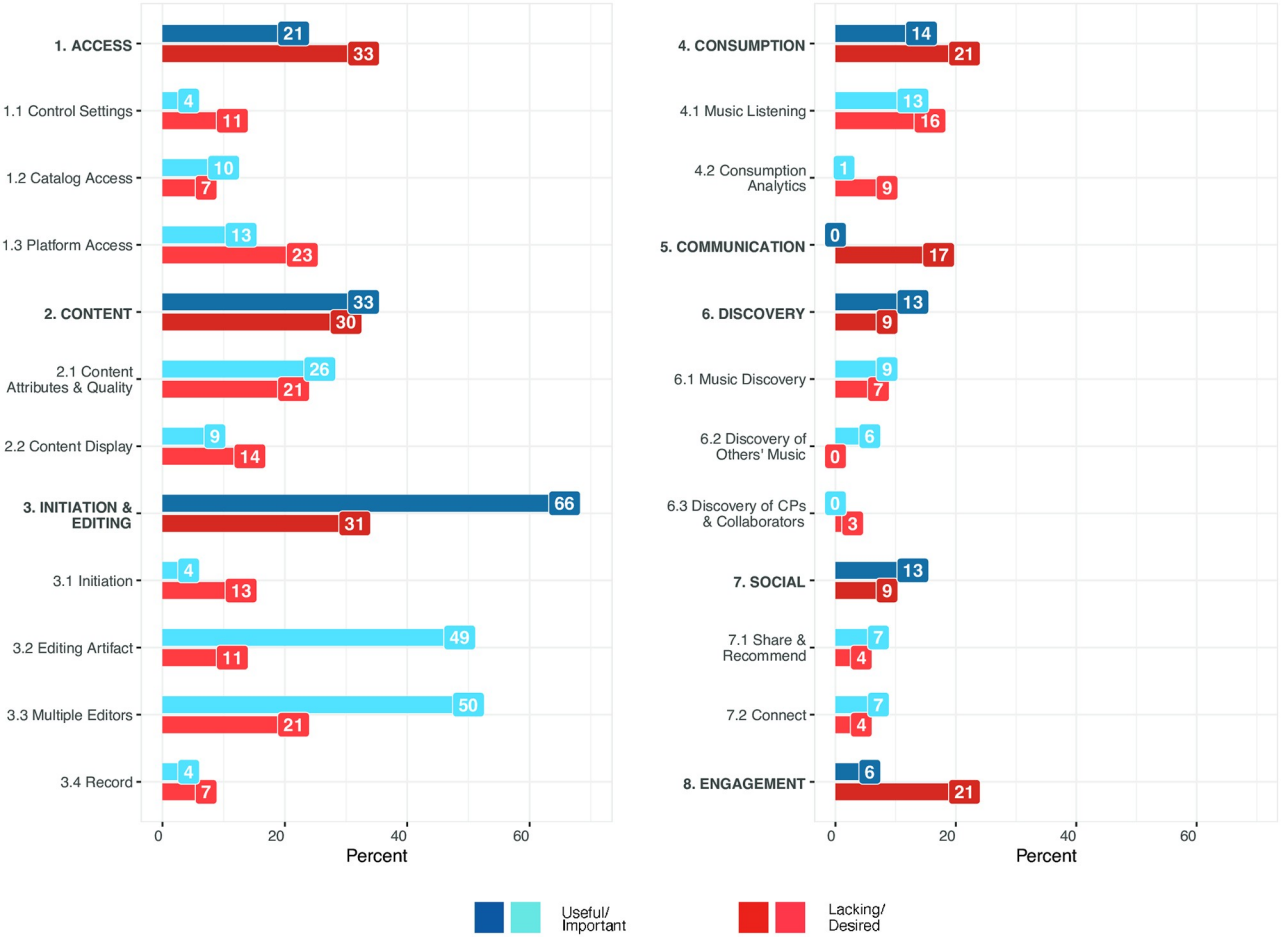
Percentages of free-text responses mentioning each of the aspects in the Codebook of Critical CP Factors that were coded (Table 1). Blue bars represent responses mentioning useful or important aspects of a codebook category, and red bars denote responses mentioning lacking or desired aspects. The high-level factors are capitalized.

Across the collection of responses, all eight aspects were referenced with regard to both their usefulness and importance (Q1) as well as their lacking or desired aspects (Q2). In addition, some responses referenced codebook categories conceptually, others mentioned specific proposed features that could improve them, and some responses were both broad and specific. Percentages of mentions for each aspect of the codebook are summarized in Table 2, and visualized with applicable sub-categories in Fig 1.

**Table 2:** Summary of the percentages of free-text responses mentioning each of the eight high-level aspects in the Codebook of Critical CP Factors. Bold text in each column indicates the top two categories for reported useful/important and lacking/desired aspects.

CP Aspect	Useful/Important	Lacking/Desired
1. Access	21%	**33%**
2. Content	**33%**	30%
3. Initiation & Editing	**66%**	**31%**
4. Consumption	14%	21%
5. Communication	0%	17%
6. Discovery	13%	9%
7. Social	13%	9%
8. Engagement	6%	21%

### Access

Responses in the Access category pertained to a user’s ability to control others’ access of the CPs, to access and navigate the platform’s music catalog, and to access the streaming platform itself. As shown in Fig 1, 21% of responses noted useful or important aspects of Access, and 33% noted aspects that were desired or lacking.

The first sub-category of Access highlighted settings to control collaborators’ CP access and editing abilities; this was mentioned positively in 4% of responses. P37 particularly appreciated the varying levels of access to playlists, including CPs: *“The public vs. private, collaborative vs. view-only settings are nice”*. At the time of data collection and writing, all collaborators on a Spotify CP had equal edit rights to a playlist. This was viewed as useful: *“Anyone”* (P3) who has access to the CP could change the playlist *“equally”* (P8).

On the negative side (11% of responses), users desired more fine-tuned control settings such as *“an optional ‘admin’ role for the playlist and approval of adding/deleting songs, as well as a fully equal-party mode instead of that option”* (P2), or for only *“certain people [to be] able to add songs”* (P37). These control settings, also referred to as *“editing permissions”* (P19), were desired in response to pain points around adding to and deleting songs from the CP: *“Hard to manage what gets [added]”* (P51) and *“someone can just delete your song and you’d never know who it was”* (P7). For such issues, users proposed specific solutions such as putting *“limits to how many songs people can add”* (P4) or allowing *“control of lead collaborators and having specific options for others”* (P64). Users also felt that control settings for the CP itself were confusing, stating that CPs *“can be confusing to share/set correct privacy settings”* (P31). Furthermore, some control settings were simply not available: *“for Spotify, collaborative playlists cannot be made public”* (P44) [2, 89].

The second sub-category contained mentions of access to the catalog of music offered by Spotify and contained in playlists. This category garnered slightly more useful/important than lacking/desired mentions. Users found helpful (10% of responses) the ease of contributing or navigating to CPs from their personal music and artist pages (P15, P24), offline access (P32), and access to the variety of music afforded by Spotify (P33). On the other hand, lacking aspects of catalog access (7% of responses) included the inability to add songs to the CP from outside of Spotify (such as P50 who desired the *“ability to add songs from the radio or out and about”*) and *“incompleteness of music availability on a given platform”* (P24).

Finally, platform access garnered the most responses among the sub-categories, primarily regarding lacking/desired aspects (23% of responses, compared to 13% useful/important). Differences in platform access restricted CP usage and sharing, and were often cited as problematic (e.g., *“it is hard to make playlists when your friends use different music platforms than you” (P21)*, *“accessibility to friends who don’t/can’t pay for the platform”* (P24)). Thus, some users wished it were *“easier to curate a playlist across many different platforms”* (P40). That participants mentioned *“having to share a password/username”* (P68) and that *“it can be difficult to make updates and collaborate through the same interface”* (P22) suggests they may have found workarounds to address the issue of platform access. Yet these and other responses, such as not being able to *“work on [the CP] from our respective devices at any time”* (P8), imply that some users may be not be aware of Spotify’s CP functionalities and may rather be co-curating through the personal playlist interface. On the positive side, useful/important aspects of platform access included general ease of access (P10, P16, P33) and that multiple collaborators can access the CPs (P10, P58, P70).

### Content

Responses in Content were related to characteristics or quality of the songs in the CP, as well as how content is displayed. 33% of responses highlighted useful and important aspects of Content, and 30% mentioned lacking or desired aspects.

For attributes and quality of Content, users mentioned useful/important attributes in 26% of responses. Users appreciated the diversity or *“variety”* (P12, P33) in the CP and the way the music of a CP represented *“selections that reflect the authentic collaboration of those who put the playlist together”* (P22) that *“can bring together many different tastes”* (P47). Others stated that the CPs enabled playlists to be formed with *“some amount of coherency in the music”* (P11) and *“continuity”* (P27), which could imply a consistent *“theme”* (P30, P54) of the music, or *“music mixes”* (P25) for specific occasions such as *“when we are together or at a party”* (P44). Lacking or desired elements were mentioned less—in 21% of responses—and included the need for more *“quality control”* (P52) of the music added, which could become an issue when *“people get carried away and add bad music and take over a playlist”* (P51). This issue—and, more broadly, CP co-curation involving more song additions than deletions—meant that CPs could become *“extremely long”* (P15) and therefore difficult to manage: *“They get long very quickly and then when they’re too long it’s annoying to shuffle through them because there’s a high chance you’ll come across a song you don’t like”* (P38). P38 additionally suggested that this could be *“a shortcoming on the collaborators’ efforts to make a NEW playlist or delete songs”*. P15 suggests *“a feature that could divide the playlist into sub-blocks that could be moved around”* to manage large quantities of music. Overall, multiple mentions of similar sentiments highlight the difficulty of maintaining a shared playlist that contains content that everyone enjoys. Interestingly, other shortcomings contrasted directly with advantages underscored in positive responses—e.g., that a CP had *“no diversity within song selection”* (P27) or was *“too focused on popular music”* (P46). As such, responses pointed to users desiring a certain makeup of the content of CPs to embody musical diversity or balance of genres. These and other reports—such as *“it is hard to get music that everyone likes”* (P30) and *“not everyone has the same or similar music tastes”* (P70)—reflect the variety of experiences pertaining to music selection that CP users may encounter.

Content display received slightly more lacking/desired than useful/important mentions (14% and 9% of responses, respectively). Users highlighted basic playlist functions not unique to CPs, such as *“being able to see the names of the songs and artists”* (P7) or (re-)ordering songs in the CP (P7, P15, P39, P47, P52). Users desired additional capabilities to display the existing content in new ways. Two common ways in which users wanted to organize CP contents were by filtering and sorting—for instance by genre (P5), contributor (P7), listening context (P15), newness (P46), or to *“distinguish recently added/temporarily added songs from more fixed songs on the playlist”* (P26). Some users also hinted that the platform could automate some of these reordering functionalities (e.g., *“filter out newer music”* (P46), *“push [top-rated songs] to the top of the list”* (P2)).

### Initiation & editing

Initiation & Editing was the most positively mentioned of the eight factors, with 66% of responses mentioning useful and important aspects; 31% of responses mentioned lacking or desired aspects. Beginning with CP initiation, 4% of responses mentioned positive aspects, such as various users stating that CPs were *“easy to use”*. But more (13% of responses) mentioned difficulty and confusion around starting or locating CPs, e.g., *“Spotify doesn’t have a very easy interface for creating collaborative playlists”* (P8), *“they are hard to configure from the get go”* (P30), *“it was very difficult for my friend and me to figure out how to create and share a collaborative playlist on Spotify”* (P53). As noted by P50, the CP creation process can also involve *“effort”* and *“technical difficulties”*.

In the second sub-category, editing the artifact refers to acts of adding, deleting, or reordering songs in the CP. This category was mentioned positively in 49% of responses, and as a lacking/desired aspect of CPs in only 11% of responses. Multiple users mentioned the general ability to edit the CP, for example *“the ability to dynamically edit the playlist over time (e.g., number position of song, deleting a song, etc.)”* (P5). The ease of *“being able to contribute any songs”* (P31) was also mentioned as important: *“The ability to easily drag songs from my playlists and add to another, preferably in volume [and] the ability to rearrange and delete songs on the collaborative playlist is important too”* (P15). Mentions of lacking or desired aspects tended to note that the CP interface is not easy to use: *“They’re just hard to use, even when everyone uses that platform”* (P40).

In the third sub-category of multiple editors (useful/desired reports in 50% of responses, lacking/desired reports in 21% of responses), responses specifically mentioned the *“collaborative aspect”* (P4) of having multiple CP editors: *“Anyone being able to contribute”* (P23) and *“everyone who has access can add their songs”* (P3), thereby enabling *“all users able to change playlists equally”* (P8). Synchronous editing was also noted as useful: *“Can both update the playlist at the same time”* (P32). In short, the *“simultaneous cloud-based collaboration”* (P24) was stated most frequently as being useful. Yet while distributed, synchronous editing is already possible on Spotify’s CPs, some responses suggested that not all users were aware this was the case; for instance, P8 felt that the CP experience would be enhanced *“if we could both work on it from our respective devices at any time”*. Other aspects of multiple editors that users found lacking included notifications (e.g., *“I would want to have notifications when other people add to the playlist”* (P14), *“a notification every time my collaborator adds a song”* (P36)). Users also desired more clarity on who added what, suggesting *“more visual cues”* (P30), such as *“a color coded flag”* (P60), to make this information more clear.

Finally, the ability to access a historical record of edits was noted as useful or important in 4% of responses, and as desired or lacking in 7% of responses: *“I can’t see the history of what was removed/added”* (P2). P7 lamented that *“there’s not really a history function and […] someone can just delete your song and [you may] not even know it was ever in the playlist to begin with”*. P26 also wished for a way to distinguish *“temporarily added songs from more fixed songs [because] you have to create another playlist to experiment”*.

Overall, co-editing capabilities are summarized in one user’s desire for CP functionalities to be more like Google Docs: *“Group editing techniques‚ similar to Google Docs would enhance such collaboration [as] it can be difficult to make updates and collaborate through the same interface”* (P22).

Multiple users highlighted the importance of these edits being made visible, for example in *“seeing which users added which songs”* (P2) and *“knowing who put what songs in the playlist”* (P7) *“and to a lesser degree, when”* (P24). Viewing these edits, especially song additions, enabled users to *“learn what music my friends like”* (P15). While at the time of data collection, songs in a CP are labeled with the handle of the user who added each one, some expressed it as a desired feature: *“I think it would be cool to see who added what although I use Spotify and that’s not currently a feature”* (P67), implying that not all users were aware of this functionality. Moreover, multiple users expressed a desire to view a more extensive history of edits, e.g., *“in case I want to undo an action”* (P2). Numerous users (14%) desired more editing visibility than just history. Multiple users wanted to receive notifications when the CP was edited, for instance as a way to know of edits without having to check the CP themselves: *“Having a way to opt-in to notifications of when people add songs to the playlist would be cool so I just know when people add new songs (and what they are) instead of not realizing that they did or having to check regularly”* (P7).

### Consumption

The Consumption aspect of CPs pertains to how users consume (i.e., listen to) the music in the CP, and to consumption analytics. Useful or important aspects of music listening, whether synchronous or asynchronous, were noted in 13% of responses. The ability to consume the CP in different contexts was seen as important (e.g., *“when we are on our own or […] when we are together or at a party”* (P44)). Users also valued the ability to access and play the CP on their own: *“I like being able to go at it at your own pace. The best feeling is when you do not add something for a while and then you check the playlist and 20 songs were added and you can just start listening to them all”* (P14). Other responses highlighted Consumption functions of playlists more broadly, not specific to CPs: *“Being able to listen any time”* (P31) and *“being able to shuffle the playlist or play specific songs”* (P7).

In lacking or desired attributes of music listening (16% of responses), a number of users expressed a desire for synchronous co-consumption: *“Being able to listen at the same time (synchronized) on separate devices in separate locations”* (P31). Users also desired *“queues”* (P1, P55)—the ability for groups *“to listen to songs with everybody contributing in real time”* (P1). Additional features to support synchronous consumption with collaborators were also requested: *“A DJ mode, where you can create transitions between songs”* (P17), a function to *“divvy whose song gets played when”* (P34), a function to *“‘hide’ songs that you personally don’t [want] to hear when you shuffle the playlist (without deleting them from the entire playlist!)”* (P38), and *“a function where I could play songs that were added to the playlist by a specific person/people, or maybe more generally, a function that let me play only songs contributed by other people and no songs I contributed myself”* (P7).

Visibility of Consumption was viewed as deficient by 9% of participants, who expressed a desire for consumption analytics. Users reported that *“it’s sort of hard to see if other people are actually listening to the playlist or not”* (P7) or *“if I’ve never listened to the song”* (P30), highlighting a lack of awareness of both others’ and one’s own CP music consumption. This desire for visibility of consumption also translated to requests for features providing *“analytics [to see] when the collaborator listens”* (P34). Only one user positively noted that this aspect already existed, stating that music included in the CPs represents *“what my family is listening to”* (P61).

### Communication

Communication refers to the expression of sentiments and thoughts among CP collaborators, e.g., about the CPs and the music contained therein. All mentions (17% of responses) noted the lack of such features, as the platform does not currently offer any such Communication channels to collaborators.

Many users desired to *“discuss the playlist”* (P15) and the songs: *“Maintaining an open, active conversation regarding new songs that should be added and old ones that should perhaps be removed”* (P27). Users also wished to *“provide feedback to each other on the songs that each collaborator adds”* (P37), which could facilitate collective decision-making such as *“2-person agreed deletion of songs”* (P4). A number of participants mentioned specific features promoting visible communication. For instance, multiple responses expressed a desire for text-based communication features, e.g., for *“individual collaborators to rate/comment on songs in the playlist, whether they like it or not, and other things”* (P7), for *“notes attached to songs”* (P24), to *“add a nice note to the top if you’re making it for/with someone”* (P67), and to provide commentary on *“why they like it [would be] nice”* (P28). Less specific suggestions included requests for features to *“comment”* or *“chat”* (P7, P15, P28, P39, P59), and to *“rate”* (P2, P7, P15, P52) or *“like”* (P39) songs.

### Discovery

Responses in the Discovery aspect pertained to learning or receiving information about music, collaborators, and even playlists. Overall, 13% of responses mentioned useful and important Discovery attributes, while 9% pointed out aspects of Discovery that were lacking or left to be desired. In terms of useful aspects for music discovery (9% of responses), CPs were reported to aid users in *“discovering new songs I would not find otherwise”* (P65) and *“new music and artists I love”* (P53). For some, CPs also acted as a portal for receiving an *“influx of new and fresh but still pertinent music”* (P19) as well as *“recommendations based on what I listen to”* (P62). Yet 7% of users also wished that platforms would do more to recommend songs for users to add to CPs, as *“the problem is that sometimes you have so many songs in your playlist that it is hard to remember which song do you think it would fit the playlist”* (P63). This could include songs that are similar to those already in the CP (P10, P45), *“songs from each other’s playlists”* (P43), and even songs resulting from *“more recommendations from AI about music that is similar [to] music and categories of music that people add”* (P20).

In the second sub-category of Discovery, users reflected positively upon the usefulness and importance of learning about collaborators’ music (6% of responses; no lacking/desired reports), for example in *“[seeing] who adds which songs so I can learn what music my friends like”* (P15) and learning *“what my family is listening to”* (P61). Moreover, P12 stated that *“I think having the ability to […] discover new songs that a person you know (such as my roommate) listens to is cool”* and P27 further notes that CPs help them better understand collaborators as people: *“What inspires them, what moves them, what do they believe in?”*

Finally, two participants noted lacking/desired aspects of discovering new collaborators and CPs; no participants gave useful/important reports. P10 expressed difficulty in discovering CPs (*“it’s kind of hard to find good collaborative playlists”*), while P14 desired recommendations for potential collaborators (*“I would love it if the application suggested WHO to create a collaborative playlist with out of my Facebook friends based on similar music tastes”*).

### Social

The Social aspect of CPs and their usage included mentions of sharing and recommending songs and playlists, or connecting with others through CP co-curation. In the present data, 13% of responses mentioned useful or important Social aspects, and 9% mentioned desired or lacking aspects.

With regard to sharing and recommending, 7% of responses reflected useful or important aspects, with users stating that CPs enabled them to *“share music”* (P12, P25, P44), have *“shared songs”* (P59), and express *“shared interests between friends”* (P41). On the other hand, 4% of responses mentioned a desire for the platform to further facilitate sharing, whether offline (P25), with a *“broader ability to share the playlist”* (P44), or even automatically by *“recommending songs from each other’s playlists”* (P43).

Social connections among collaborators were also noted. On the positive side (7% of responses), users mentioned that encountering others’ contributions to a CP can reflect *“relatability”* (P46), *“shared interests between friends”* (P41), *“the authentic collaboration of those put the playlist together”* (P22), and *“lots of heart in what songs that person puts on the playlist”* (P27). P14 reports that CPs can make them *“think of the person that you made it with”*. Yet 4% of users found the social aspect lacking (P48) and, as noted in Discovery above, that the platform could make better use of users’ social connections on other platforms such as Facebook to suggest potential CP collaborators (P14).

### Engagement

Engagement, the final category in the codebook, pertains to mentions of longevity, awareness, and intrinsic process of CPs, as well as feelings surrounding their use. Only 6% of responses mentioned useful aspects of Engagement, i.e., the enjoyment of creating, curating, and consuming CPs. For instance, P19 noted the *“humor with which you can approach the process”*, while P63 noted that *“everyone can participate regardless of their music taste”*.

Meanwhile, 21% of mentions pointed to lacking or desired aspects of Engagement. Hinting at CPs falling short of their desired longevity or sustained engagement over time, one user stated that *“they are only used for a short period of time”* (P18). Another lamented that collaborations around CPs were not as engaging as they would like: *“I don’t feel as engaged to work on them. [CPs] have been created with the intention of being collaborative, but no one else ends up adding or curating them”* (P35).

Overall, responses in this category highlighted a desire for greater engagement with CPs—from themselves as well as others—coupled with a lack of awareness of current engagement. Many users wanted to know whether others were engaging, regardless of whether it was through edits or consumption. For one, P35 felt that visible edits, as operationalized by notifications, would motivate greater engagement: *“I think that notifications indicating when things are adjusted and changed would help to encourage more interaction”*. Such notifications may even *“[get] a group to regularly contribute”* (P68). Others wished for notifications in the *absence* of engagement, as CPs *“often get forgotten/not added to after a while”* (P50). Here, users suggested that CPs *“should be subtly promoted on the streaming platforms”* (P50) and showed desire for *“a notification from the playlist platform to check out the collaborative playlist when it starts becoming inactive”* (P41). One user even noted that *“not everyone is aware of collaborative playlists”* (P62), suggesting that awareness of and engagement with CPs could be improved on even a broader level.

In terms of the intrinsic process of CP usage, one user suggested *“[making] it more ‘fun’”* and providing more of a *“‘social platform’ appeal”* (P48). Finally, with regard to feelings—which could be personal or inter-personal—participants mentioned anxieties around sharing music (e.g., *“afraid to add songs that others may not like”* (P9)), challenges related to disparate musical tastes (e.g., *“hard to get music that everyone likes”* (P30)), and unwanted behaviors among collaborators (e.g., getting *“carried away”* and *“tak[ing] over a playlist”* (P51)).

## Discussion

We surveyed users of collaborative playlists and conducted thematic analysis of open-ended text responses to better understand what aspects of CPs and their usage are considered useful, important, desired, and lacking. Based on responses from 70 participants who had used CPs on Spotify, our analyses uncovered eight high-level aspects of CPs and their usage: Access, Content, Initiation & Editing, Consumption, Communication, Discovery, Social, and Engagement (Table 1).

At the time of data collection, CPs were essentially “personal-plus” artifacts—that is, playlists with co-editing functionality and indication of who contributed each song in the playlist and when. Unsurprisingly, participants confirmed that these basic functionalities are useful aspects of the CP experience. However, results also suggest that there is more to be done to support user needs around CPs. Our results, interpreted in relation to prior literature, collectively point to numerous lacking or desired features in each of the eight CP factors, highlighting opportunities for engaging users further in CPs. From these insights, we offer design implications for CP platforms that can inform the design of other collaborative platforms.

### Design of CP functionalities ought to be informed by users’ purposes for engaging in CPs

Three of the eight factors of CP usage that emerged from our inductive coding process—Content, Discovery, and Social—align with the Practical, Cognitive, and Social purposes, respectively, of the CP Framework [1]. Each of these factors was mentioned with more emphasis on useful or important aspects over lacking or desired aspects, but only by a slim margin (Table 2). This suggests that the purposes for which people engage in CPs are largely fulfilled in practice, though there is room for streaming platforms to improve user experiences to serve CP purposes further. These categories also echo reports from social music prototype research, underscoring our findings that social curation and consumption empower users to achieve music goals.

Text responses from the present sample highlighted positive and negative aspects of Content that emerge during CP co-curation. Aspects of Content are noted in previous work as well. For instance, Lehtiniemi et al. reported that users of their prototype “thought more carefully about the content they added to the service since they were more socially aware of other users”, creating “positive pressure for content creation” [52]. But, similar to our findings, prototype users have also reported challenges of too much music or bad music [48, 49]—the latter of which could compel users to abandon the social platform altogether if too many bad songs were presented in succession [49].

Responses positively implicating the Discovery aspect of CPs, especially around discovery of new music and discovery of others’ tastes, also align with past research. With Jukola, for example “one of the key values of the system was learning about new music” [45]; this was also reported by users of tunA [47] and Push!Music [48]. In the present study, users reported multifaceted perspectives of Discovery, reminiscent of the potential of social prototypes to help users “discover new music through their friends, and [make] new discoveries about the friends as well” [49]. Finally, discovery of new collaborators—a wish expressed in the current sample—was also reported in a number of prototype studies [44, 47, 51].

Responses mentioning Social aspects of CP usage reflected music sharing and recommendation, as well as social connections that are enforced by CPs and collaborations. Past prototype studies similarly report that users enjoyed sharing music through such platforms [48] and that doing so can strengthen relationships [49]. In addition, the potential for CPs to heighten social outcomes through collective reminiscence has been noted in prior work, e.g., in posted historical records of “winning” Jukola songs [45] and synchronous listening while engaging over social media [41]. However, similar to present findings, past studies report that music contributions alone do not serve all of users’ social needs (e.g., users of Social Playlist were “not able to interpret the states of others by listening to the music itself” [49]). Given that Social purposes for engaging with CPs have been found to be just as important as Practical (e.g., Content) purposes [1], features to fulfill Social aspects of CPs may also greatly enhance users’ experiences.

As reported in the original CP Framework study [1], our findings show that multiple CP Framework categories can be implicated at once. For example, P53’s report that multiple contributors *“[allow] a playlist to have diversity and leads me to discover new music”* implicates both Content and Discovery. One category does not subsume the other but can influence it: Content can be referenced without implicating Discovery (e.g., when referring to the theme or quality of the CP or its songs) and Discovery without implicating Content (e.g., with regard to Discovery of collaborators’ tastes or collaborators/CPs themselves). In introducing the CP Framework, Park and colleagues also note that the CP Framework categories can “facilitate one another” [1]. Music prototype research echoes this point: Bassoli and colleagues write that successful Discovery can bring about Social benefits, e.g., “creat[ing] new meaningful social connections […] having a communication channel to start a ‘virtual conversation’” [47]. Our current findings confirm this point and suggest further connections across factors. For example, improved Communication could improve both the Social and Content aspects of CP usage, opening additional channels for Discovery; and improved analytics and visualizations of Consumption could help collaborators improve CP Content, for instance through removal of songs that no one is listening to.

*Implication.* As current CP functionalities provide the bare minimum for collaboration, opportunities exist for CPs to better serve users in aspects reflecting CP Framework purposes [1]. We suggest that consideration of platform features be guided by users’ purposes for engaging with CPs, with features evaluated against how well they meet the critical factors identified from this work. For example, including indicators of social presence on CPs can lead to more serendipitous opportunities for connection and avenues to reminisce together, and thereby better fulfill users’ Social purpose of CPs. Including coherence/diversity metrics or collaborators’ contribution proportions for each CP as a way to create and maintain quality CP Content can help users achieve their Practical purpose. Providing characterizations of the music contained in the CP or the music tastes of collaborators can also help Discovery. As such, expanding upon current functionalities with the CP purposes for which they are designed in mind can help to better support users.

### CP users seek finer controls when interacting with CPs

Control is an important factor in personal playlist curation [18, 90, 91] and arguably even more so for CPs, as there are also social dynamics at play. A common thread amongst many of the themes that emerged in participants’ text responses was their desire for finer control—for facets of access, in the makeup of CP content, of the editing process, and during consumption. Starting from these factors of Access, Content, and Consumption, CP designs could benefit from insights from control settings of other collaborative platforms.

#### Both egalitarian and hierarchical collaboration dynamics are desired

Controlling others’ access and editing actions has been highlighted in admin-type functionalities in music prototypes [48, 51]. Many responses in the present study similarly expressed a desire for greater control in CPs—of fine-tuned controls of who gets access to the CPs, and what levels of contributions they can make within the CPs. This is what one user may have been alluding to by stating *“Group editing techniques‚ similar to Google Docs‚ would enhance such collaboration”* (P22).

Yet it is not simply a range of access and editing controls that users desire. In fact, mentions of lacking fine-tuned control features allude to two types of CP collaborations for which participants desire to receive support: *Egalitarian* and *hierarchical*. Participant responses suggest that the current design of CPs affords egalitarian collaborative experiences: As mentioned by several participants, enabling every collaborator to edit the CP *“equally”* and to represent their music taste with such a spirit were important and appreciated. Moreover, features in the egalitarian spirit were desired, including voting mechanisms and group decision-making. This is aligned with prior findings. Past social music systems have discussed attenuated forms of control over music content, exerted through forms of group influence such as ratings [49], voting [45], or specified preferences [42].

However, some also wanted control settings that would enable certain participants to have more control than others. This suggests a desire for some hierarchy within the collaborating group, and a lack of support for such a configuration. Prior work on collaborative mood music curation (MoodPic) confirms that both of these desires may co-exist among users of a single platform: “Some participants were requesting ways to control the content of some specific playlists, where other playlists could remain open for additions […] however, some did not want only the creator of the list to have all the control, but instead sharing the control to all of the users of the collective list” [51]. Echoing prior work, current participants mentioned desiring both types of collaborations, showing that the type of collaboration of the CPs depends on the context and not just the individual.

*Implication.* Two kinds of collaborations that are present in team literature [92] also exist in the context of CPs. CP platforms need to recognize these two kinds of collaborations that users are seeking and/or carrying out, and support both. Designing control elements of access to and edits on CPs is necessary to ensure satisfactory CP experiences of both types. Contrary to the notion of control itself, these features can foster a sense of democratic collaboration and give users free rein to interact with the CP, bringing greater engagement and comfort to the collaboration. But they must be considered alongside potential tradeoffs between control versus active engagement [52, 93]. As explored in prior work [8], further exploration of specific features already implemented in other collaborative platforms can also help improve upon the current CP experience.

#### Finer editing and organizing tools can help improve CP manageability

While Initiation & Editing was one of the factors mentioned most positively, participants’ desire for more tools to organize content reflects a common occurrence in CPs, which P15 stated aptly: *“Sometimes the playlists are extremely long”*. This is due in part to collaborators *“not deleting songs”* (P27), which may reflect hesitation and discomfort arising from perceived ownership of songs in CPs, as noted by Park & Lee [9]. P38’s desired functionality to *“‘hide’ songs […] without deleting them from the entire playlist”* is one solution. Certainly the filter and sort functions noted by multiple users are another way of addressing this problem of “long” playlists. In addition, as P26 noted, separating songs based on pre-articulated and agreed-upon criteria, such as *“recently added/temporarily added”* from *“fixed songs”*, and having a designated space in the interface for such songs, may be helpful. These features could also prove to be useful in managing personal playlists, which are also known to have size issues [94, 95].

*Implication.* Manageability of playlists has long been an issue, even predating CPs. Yet this issue is exacerbated in the context of CPs for various reasons including division of control amongst collaborators and difficulties of deleting songs due to social norms and pressures. To the latter point, enabling a *“2-person agreed deletion”* (P4) functionality is one option. As noted in prior CP work [9], enabling comfort felt in deleting songs in the CPs as well as contributions by other collaborators by providing communication mechanisms can mitigate this problem. Implementing such designs can lead to more positive experiences for the users navigating the CPs, which most likely contain more diversity of music that requires breaking down into parts that are more manageable. By addressing this, the hindrances in engaging with the CPs may be reduced, and enable greater engagement with CPs and amongst collaborators. This can increase the chances of CPs becoming more successful, as engagement has been found to be an indicative factor of CP success [8].

#### Post-production experiences need to be considered and require finer controls

One aspect of CPs distinguishing them from many other online collaborations is the ensuing consumption by collaborators during and even after the collaboration. In contrast to *production-based* collaborations—which have “the intention of producing a final product to be published or released” [9]—collaborating on playlists can also be rather *consumption-based*. Our present findings with CPs highlight the need to accommodate a wider range and variety of such consumption practices.

As consumption is very much part of the playlist experience in general [18], users desire control of this aspect. Prior work shows the importance of control for music curated for social contexts, such as parties and road trips [30, 31]. Similarly, participants also desired finer controls in consuming CP music—of being able to *“create collaborative queues […] in real time as they are added”* (P1), listen to them synchronously in different locations (P31), *“play songs that were added to the playlist by a specific person/people”* or by everyone else other than the participant themselves (P7), and create transitions between songs (P17). Such a desire for finer controls for consumption may be more difficult to infer from existing collaborative platforms, as many are for production-based collaborations.

*Implication.* Users’ experiences of CPs go beyond co-creation to consumption as well, whether individually or as a group. Participants’ proposals to use filtering, sorting, and skipping to personalize the consumption of a shared playlist are some ways in which consumption of CPs can be considered. Implementing such features can help collaborators become better acquainted with the music others have added, e.g., as they filter and listen for a particular collaborator’s song additions. This can in turn increase awareness of each others’ music tastes and possibly help them to recommend music that is better aligned. Synchronous co-listening among geographically distanced collaborators, in the same spirit that distanced friends and partners can watch streaming video together [96, 97], may also help collaborators connect better, as CPs already do [1]. CP designs providing better experiences for users’ consumption can further inspire design for co-curation and consumption (i.e., post-production) experiences of other collaborative artifacts as well.

### CP users want to better understand their collaborators

Visibility of others’ interactions is known to be essential for successful collaborations on online platforms [98]. One way in which CPs differ from other online collaborations is that the end product—that is, the playlist—may involve a greater degree of repeat consumption (e.g., for entertainment) [8]; as mentioned earlier, it can also be not only production-based but also consumption-based. Consequently, and as suggested by the present data, collaborative interactions with a CP should consider how users can gain awareness of their collaborators’ editing and consumption actions.

Moreover, the desire for greater awareness expressed both here and in user interviews regarding successful CPs [8] suggests that collaborators want to better understand one another. The fact that there are multiple contributors acting upon the CP introduces the possibility of missing out on both the *event* of modification (the modification itself, e.g., a song added or deleted between between visits) and what a modification *signals* (the motivation/meaning behind the modification, e.g., why a collaborator added a song). Users also reported that it is difficult to know whether or how collaborators enjoy or engage with songs in a CP. In other words, current “personal-plus” implementations do not adequately communicate the information that users want. The desire to access and contribute information—from how many times a collaborator has listened to a song, to a “like” or comment—highlights a current gap in communication between collaborators.

#### Of edits and consumption (what others do)

Wishing to access the version history of a CP shows a desire to understand not only a playlist’s content but also its process, a sentiment reflected in past work [48]. The consequent ability to undo changes may also give collaborators more psychological safety or comfort to edit a CP. Users’ perceptions of functions are also highly critical. For example, P67 was not aware of an existing functionality that allowed users to see *“who added what”* within a CP. This perception is critical to the user’s CP experience as it also influences how they interact through the CPs. Hence we need to first understand why such perceptions exist and then address them in designing features. While further investigation is needed to accurately explain why, we speculate that this particular user perception may arise because the platform’s desktop application is showing usernames only, or the user interacts with CPs only through a mobile application that does not make this immediately visible. Furthermore, having this knowledge and awareness of who made which edits in a CP—whether gained by accessing the playlist or by receiving a notification—can help users achieve a sufficient level of workspace awareness [63, 64] and improve the collaboration. In fact, Communication, too, could indirectly enable users to know whether collaborators have added to or listened to a CP, and provide insight into their feelings about a given track.

Users also wanted to know how collaborators were consuming CP content. This user need has been highlighted in past music prototype research, and motivations can range from curiosity [48, 51] to wanting to better understand others’ musical tastes [50]. Past studies have also reported that users feel a sense of accomplishment or motivation from knowing that others are listening to content that they contributed [45, 49, 51]. Consumption analytics can also promote engagement, as “information on how many users have listened to their playlist […] was said to motivate playlist creation even more” [52]. We surmise that knowledge of CP consumption behaviors could also help users identify songs to add or delete. Finally, past works have noted that such transparency can strengthen interpersonal dynamics [49, 51], and that a better understanding of collaborators’ tastes (by knowing what they are doing) can diminish anxiety around adding songs [50].

However, surfacing consumption behaviors may bring about less-desired outcomes as well. Self-consciousness over how others view one’s music tastes (e.g., as reflected by sharing and consumption) has been noted in past studies [28, 49, 50]. Users have expressed contradictory wishes between wanting to know what others are doing, but not wanting their own actions to be tracked [93]. Along these lines, the extent of transparency in CPs could fundamentally change how users listen. With Pocketsong, for example, Kirk et al. reported that users changed their listening habits as a result of their listening record being visible, “shap[ing] their listening not just for their consumption but explicitly for the consumption of others” [50]. Therefore, care is needed to strike a balance between requests for transparency, and users’ privacy needs and comfort levels.

*Implication.* Users seek more information on how CPs are edited and consumed. We recommend that platform designers carefully consider how a feature will balance needs for both transparency and privacy, which will likely vary by activity and user. This might entail providing granularity in transparency settings, e.g., not showing counts of certain songs played in the CP, or not presenting songs only listened to in private listening mode when the system suggests songs to add to a CP. Or, users could select the extent of personal data they share on a per-playlist basis, as in Beierle et al. [99]—perhaps in a reciprocal fashion, where the visibility a user grants others is the visibility granted to that user. Learning from research on change awareness [60] with mechanisms such as those for version control [54] to increase granularity in visibility of editing would be helpful. Such awareness features will help to motivate collaborators toward greater engagement, psychological safety, and group dynamics in CPs, while addressing worries around privacy and impression management.

#### Of communication (what others think and feel)

The act of music sharing is a noted form of social interaction [50], yet the social music behavior of conversing around music is at risk of being lost in the digital age [100]. Our present results indicate that users seek additional communication features such as likes, ratings, and comments. These expressions are known to improve collaborations over online platforms [101] and provide a sense of copresence on other music platforms such as SoundCloud [35]. A number of benefits are noted in social music prototypes as well: Additional means of communication facilitate richer interactions [50] and music discovery [45]; let users know whether others liked their song recommendations [48]; enable users to explain selections, potentially reducing misinterpretations [49]; and help to form or strengthen social relationships [47, 49]. These results are corroborated by findings that users would feel more comfortable about taking actions on CPs—particularly uncomfortable ones—if given a channel to communicate [9]. Spotify previously offered a chat feature (not specific to CPs) that allowed users to exchange messages; however, this functionality was “removed […] due to low overall use combined with the resources necessary to keep it running well” [102]. While a regular chat functionality between all users may not have been feasible or useful, providing a communication channel for collaborators to explain their thoughts and express their feelings could improve the CP experience.

On the other hand, music prototype users have expressed concerns that comments would “fill the whole service with irrelevant content” [52], and that communication features would displace music as the central focus [51]. Finally, prototype users have reported a risk of feeling bad about their content contributions if others were to respond to them negatively [51].

*Implication.* Currently, users rely upon only the *events* of each others’ song additions, deletions, and reorderings—if they are even noticed. Hence, interactions around CPs lack social cues to help collaborators better understand each other. Enabling CP users to communicate and exchange sentiments—whether through comments, likes, ratings, or alternate features altogether—can further enhance engagement with playlists and the music platform. However, prior work has found that even prevalent online actions, such as clicking a “like” button, can have various connotations and be misunderstood [103]. Therefore, implementing features with more nuance (e.g., part of song liked, reason for liking a song) may be helpful, and could help inform other collaborative platform designs. It will be imperative, however, that communication features do not take over the music. As with edit records and transparency, optimal design settings will likely differ according to the sentiment and the user. Enabling situated exchange with annotations [55] to address communication may a starting point. What the platform should do with the sentiments also remains an open question. For instance, recent research has shown that people react differently to positive versus negative feedback in group playlist curation [22]. Therefore, continued research will be needed to understand how platforms can best surface and act upon collaborators’ expressed sentiments.

### Users seek active engagement with CPs and look to system interventions to support their engagement

An active community is known to be critical to the success of social music platforms [52] and of collaborative content in general [67]. In our study, Engagement was largely a lacking or desired aspect of CP usage. For instance, users reported that they or their collaborators did not engage enough in the CP, even though there was a desire to do so. Some suggested that even if they were too busy to pay attention to CPs, they would like to. Others felt engaged, but felt that others were not matching their level of involvement. Park & Lee suggest that issues of perceived ownership can also affect CP engagement [9]; hesitations around editing music in CPs as they pleased could hinder users from further consuming them. This underscores participants *wanting* themselves as well as others to engage in CPs. Whether this desire is due to collaborators themselves not being able to engage as actively as they would like, or misaligned expectations and wanting others to contribute more, it needs to be addressed.

Engagement was an underlying, and perhaps also unifying, factor across all of the codebook categories. First, lacking or desired features in other categories can cause Engagement to suffer. For instance, aspects of Access—such as inadequate control settings, or insufficient platform access across collaborators—can hinder CP collaboration at a foundational level, while bad or excess content can cause people to disengage with a CP [49]. The two direct interactions with CPs—Editing and Consumption—combined with a desire for greater visibility of both—also point to CP users’ desire for higher engagement with the playlists. For instance, many users’ desire for notifications, particularly when songs are added, hint at their untapped potential for Engagement. As noted above, a lack of affordance for Communication among collaborators is also a missed opportunity to encourage ongoing interactions with a CP, and can lead to suboptimal CP content and misunderstandings among collaborators. Not knowing what others are doing or thinking inhibits both the procedural aspects of CP curation and the music-related conversations and interactions that are a critical piece of social music behavior [21]. Inadequate Discovery of CPs or collaborators can dampen CP engagement: Just as the unprecedented scale of content accessible on streaming platforms can be overwhelming [20, 21], finding the right playlists and collaborators (e.g., given vast followership on social media) for co-curation is also of great import. Finally, Social aspects are hindered when users do not participate because they do not know what music to share, or because the CP does not serve to establish or deepen connections with collaborators.

At the same time, successful Engagement can positively feed back into other aspects of CP usage. For instance, an active community of collaborators can create “positive pressure for content creation” [52], while greater Engagement with CPs can bring about more contributions, which would enhance Discovery. In turn, understanding others’ tastes better through their contributions to the CP, or through consumption analytics or Communication (such as comments or likes), can encourage others to contribute more and facilitate positive Social aspects of CP usage. This in turn can further drive Engagement, perpetuating the positive cycle or “snowball effect” of successful collective content [67]. Active participation is also noted as a predictor of CP success [8]—i.e., active Engagement would beget even greater CP Engagement. Features that return control back to individual users within a group setting have the potential to decrease user anxiety (because they can perform actions without changing the public document)—an issue that has been noted previously [50, 52]—and also increase Engagement (e.g., if a user is especially interested in newly added songs or songs contributed by a specific collaborator). Finally, heightened Engagement would provide rich information to the platform, whether implicit or explicit, which could inform further development of social music capabilities.

Since data collection, some of these critical factors have been addressed in commercial platforms through new features. For instance, Spotify has released Tastebuds, which aids Discovery as users can “explore the music taste profiles of their friends” [104]. Updates to the CP enable users to more easily invite new collaborators and display avatars in front of each contribution [16]. Listening Together, an interactive globe displaying users who stream a song at the same time, was also released by Spotify in spring 2020 [105], as was Group Session, a shared queue for synchronous listening [106]. Both of these latter functionalities touch upon aspects of synchronous co-consumption of music—yet only a few users in the present study expressed a need for synchronous listening functionalities, at least in the context of CPs. Therefore, it is also important to note that some aspects of CP usage call for more urgent platform development than others. In particular, control settings and platform access (in Access), ease of creating CPs (in Initiation & Editing), consumption analytics (in Consumption), Communication, and Engagement are all disproportionately reported in relation to lacking or desired features, while other aspects already better serve users’ needs.

*Implication.* Factors that facilitate successful engagement with CPs differ from those of personal playlists. That the burden of notifying others about a CP is currently placed on the user means missed opportunities for interactions, which in turn can bring about indifference and a lack of reciprocation in the CP. This is especially unfortunate given that a few bad collaborative experiences can discourage CP usage, even if users were initially optimistic and excited about it [49, 107]. Thus, by supporting greater interaction with CPs, platforms can easily augment users’ experiences. Determining appropriate methods of promoting Engagement—e.g., opt-in notifications of added songs or when a song was played—will be necessary. Providing new ways to engage with CPs and other collaborators (Communication), promoting Engagement via notifications of edited playlists of reminders of forgotten ones, or helping users engage (e.g., by suggesting songs to contribute to a CP) may be ways to do so. Yet features for heightened Engagement need not be completely reimagined; existing methods could be repurposed. As one example, music recommendation systems (MRS) are popular and even necessary to help streaming platform users navigate millions of songs [5, 108]. However, MRS are known to bias listening habits, reducing diversity of consumption and favoring popular songs and genres [109, 110]. Therefore, further promotion or development of features suggesting songs for users to add to playlists, such as the one already available in the Spotify CP interface, should consider whether such features are useful to CP users in practice (recent research suggests they are not [8]) and, if used, how such system interventions impact other factors such as Content and Discovery. Furthermore, AI and recommendation systems designed to help individual users navigate music [5] could also be applied to finding potential CPs and collaborators [47]. In all, platform designers should critically examine ways in which current or planned functionalities facilitate or hinder user Engagement with the CP.

## Future works and limitations

The Codebook of Critical CP Factors offers a holistic framework detailing aspects of CPs that users find important. Future work can apply the codebook to study different types of CP users or use cases. For example, personas are known to provide a useful framing for investigating perceptions and behaviors of different types of users [66, 87]; if CP user personas also exist, they may implicate aspects of the codebook differently. Other studies could investigate impact of culture, collaborator group size and group dynamics, and CP purpose on codebook category membership. While the current work investigates needs of individual users, future work can also consider group behaviors, such as collective decision-making and reaching consensus together. Finally, we look forward to studying real-world outcomes of the new Spotify features mentioned above [16, 104–106], and hope that commercial platforms will additionally prioritize the most critical CP factors identified here.

While Spotify usage was not an eligibility requirement of this study, all participants were Spotify users of CPs; this likely influenced the codebook categories. We recognize that the extent to which codebook categories are referenced as useful/important or lacking/desired beyond the current sample will likely vary according to streaming service. For instance, CPs on Spotify cannot not be public [2, 89]; but a CP created on Deezer at the time of writing can only be public [111], and CPs on YouTube can be unlisted or public [112]. Usage of CPs that are also public could raise issues around privacy that were not noted by the current sample. On the other hand, user needs that are less platform-specific—such as the desire to communicate with collaborators or increase CP engagement—may generalize better. In all, while current findings are informed by usage of a single platform, the categories of user needs presented here can still inform CP platform design. Future research can specifically target users of other platforms to clarify the role of platform affordances in serving CP users’ needs.

The codebook was derived from user responses reflecting CP usage prior to COVID-19, and CP usage and user needs may have changed since the onset of the pandemic. However, we posit that our findings—reflecting long-term usage of CPs—serve as a valid representation of user needs. Moreover, the codebook, reflecting pre-COVID usage, can serve as a useful basis for comparison in studies seeking to determine how musical practices—including usage of streaming platforms and social music curation—have changed in an era where virtual collaboration has become a necessity.

Finally, lacking or desired features reported by CP users are necessarily speculative rather than experience-based, and explicitly stated rather than implicitly observed. Even so, the extent to which our codebook categories align with past research on CPs, social music prototypes, and collaborative systems speaks to their relevance for platform design implications. Future work should include direct observational and field studies to confirm whether proposed features bring enjoyment in practice or give rise to unforeseen challenges.

## Conclusion

Collaborative playlists (CPs) facilitate social music practices in the age of streaming. Yet we lack a complete understanding of which aspects of CPs promote a positive user experience, and which are lacking. To address these questions, we conducted a survey in which users with real-world CP experience on Spotify reported on these facets of CPs and their usage. Based on thematic analysis of free-text responses, we created the Codebook of Critical CP Factors, comprising eight aspects of CPs and their experience that users consider important: Access, Content, Initiation & Editing, Consumption, Communication, Discovery, Social, and Engagement. Based on the responses and the extent to which they implicate each factor, we could assess the level of importance these aspects have to users, as well as their specific benefits, obstacles, and suggested solutions. For instance, participants considered Initiation & Editing (66%) and Content (33%) aspects to be most useful, while Initiation & Editing (31%) and Content (30%) were concurrently among the most lacking and desired aspects, along with Access (33%). These percentages of mentions cue us in to which factors ought to be prioritized in order to enhance the CP experience from the user perspective.

Participants’ reports of system needs reflect in-situ, long-term usage of a widely used commercial music streaming platform. Synthesizing these results alongside findings from prior works on social music prototypes and other collaborative platforms, we have derived design implications for CPs and their functionalities. The codebook and design implications together build a better understanding of CPs and contribute toward a more socially connected music experience, especially in this time of social and physical distancing. The user-centered design implications not only enable streaming platforms to prioritize certain aspects over others, but also have the potential to provide users with a more collaborative and enjoyable CP experience. In all, these contributions highlight the potential for streaming platforms to serve the needs not only of individuals, but of users consuming music together.

## Supporting information

S1 Table Free-text responses. Anonymized free-text responses (with only participant numbers) from 70 real-world users of collaborative playlists, who reported on collaborative playlist features they found most important or useful (Q1) as well as observed shortcomings and desired features in collaborative playlist platforms (Q2).
(CSV)
